# Stability and Hopf Bifurcation for a Delayed SLBRS Computer Virus Model

**DOI:** 10.1155/2014/373171

**Published:** 2014-08-17

**Authors:** Zizhen Zhang, Huizhong Yang

**Affiliations:** ^1^School of Management Science and Engineering, Anhui University of Finance and Economics, Bengbu 233030, China; ^2^Key Laboratory of Advanced Process Control for Light Industry (Ministry of Education), Jiangnan University, Wuxi 214122, China

## Abstract

By incorporating the time delay due to the period that computers use antivirus software to clean the virus into the SLBRS model a delayed SLBRS computer virus model is proposed in this paper. The dynamical behaviors which include local stability and Hopf bifurcation are investigated by regarding the delay as bifurcating parameter. Specially, direction and stability of the Hopf bifurcation are derived by applying the normal form method and center manifold theory. Finally, an illustrative example is also presented to testify our analytical results.

## 1. Introduction

For the purpose of studying the prevalence of computer viruses and posing effective strategies of controlling their spread across the Internet and considering the similarity between the propagation of computer virus across the Internet and that of biological viruses across a population, many scholars have proposed computer virus models by properly modifying some epidemiological models [1–6]. In [2], Han and Tan proposed a computer virus model using an SIRS model and investigated the local stability and Hopf bifurcation for the endemic state of the model. In [4], Mishra and Pandey proposed an SEIRS model for the transmission of worms in computer network through vertical transmission and they obtained the threshold value determining whether the worms die out. All the models above assume that an infected computer in which the virus resides is in latency and cannot infect other computers.

Recently, Yang et al. [7] proposed the following SLBRS computer virus model by taking into account the fact that a computer immediately possesses infection ability once it is infected. Consider

(1) dS(t)dt=  μ−βS(t)(L(t)+B(t))−μS(t)+αR(t),dL(t)dt=  βS(t)(L(t)+B(t))−εL(t)−μL(t),dB(t)dt=  εL(t)−γB(t)−μB(t),dR(t)dt=  γB(t)−αR(t)−μR(t),

where *S*(*t*) denotes the percentage of computers that have no immunity at time *t*; *L*(*t*) denotes the percentage of infected computers that are latent at time *t*; *B*(*t*) denotes the percentage of infected computers that are breaking out at time *t*; and *R*(*t*) denotes the percentage of uninfected computers that have temporary immunity at time *t*. *μ* is the constant rate for the external computers connecting to the Internet and that for the internal computers disconnecting from the Internet. *β* is the rate of contact for the virus-free computer. *α* is the rate coefficient from class *R* to class *S*, *ε* is the rate coefficient from class *L* to class *B*, and *γ* is the rate coefficient from class *B* to class *R*. Yang et al. [7] studied global stability of the model by constructing a Lyapunov function for the model.

It is well known that there may be a time lag when the computer uses antivirus software to clean the virus and computer virus models with time delay have been studied by many scholars [1, 2, 6, 8–15]. In [8], Feng et al. investigated the Hopf bifurcation of delayed SIRS computer virus propagation model by regarding the latent period of computer virus as a bifurcation parameter. In [10], Liu investigated the Hopf bifurcation of a delayed SEIQRS model for the transmission of malicious objects in computer network by regarding the time delay due to the temporary immunity period as a bifurcation parameter. To the best of our knowledge, few papers deal with the research of Hopf bifurcation of system (1). Motivated by the work above and considering that there may be a time lag when the computer uses antivirus software to clean the virus, we incorporate time delay into system (1) and investigate the Hopf bifurcation of the following delayed SLBRS system:

(2) dS(t)dt=  μ−βS(t)(L(t)+B(t))−μS(t)+αR(t),dL(t)dt=  βS(t)(L(t)+B(t))−εL(t)−μL(t),dB(t)dt=  εL(t)−γB(t−τ)−μB(t),dR(t)dt=  γB(t−τ)−αR(t)−μR(t),

where *τ* is the time delay due to the period that a computer uses antivirus software to clean the virus.

The main purpose of this paper is to investigate the effect of delay on system (2). This paper is organized as follows. In Section 2, sufficient conditions for local stability and existence of local Hopf bifurcation are obtained by analyzing the distribution of the roots of the associated characteristic equation. In Section 3, direction and stability of the Hopf bifurcation are studied by applying the normal form theory and center manifold theorem. In Section 4, some numerical simulations are presented to verify the theoretical results.

## 2. Stability and Existence of Local Hopf Bifurcation

By a simple computation, we can conclude that if *R*_0_ > 1, then system (2) has a unique positive equilibrium *D*_∗_(*S*_∗_, *L*_∗_, *B*_∗_, *R*_∗_), where

(3) S∗=  (ε+μ)(γ+μ)β(ε+γ+μ),L∗=  μ(α+μ)(γ+μ)(1−1/R0)(α+μ)(ε+μ)(γ+μ)−αεγ,B∗=  εL∗γ+μ,  R∗=γB∗α+μ

and *R*_0_ = *β*(*ε* + *γ* + *μ*)/(*ε* + *μ*)(*γ* + *μ*) is called the basic reproduction number.

The linearized system of system (2) is

(4) dS(t)dt=  a1S(t)+a2L(t)+a3B(t)+a4R(t),dL(t)dt=  a5S(t)+a6L(t)+a7B(t),dB(t)dt=  a8L(t)+a9B(t)+b1B(t−τ),dR(t)dt=  a10R(t)+b2B(t−τ),

where

(5) $$\begin{array}{c} a_{1}=-\mu -\beta \left(L_{*}+B_{*}\right),\;\;a_{2}=-\beta S_{*},\;\;a_{3}=-\beta S_{*},\\ a_{4}=\alpha ,\;\;a_{5}=\beta \left(L_{*}+B_{*}\right),\;\;a_{6}=\beta S_{*}-\varepsilon -\mu ,\\ a_{7}=\beta S_{*},\;\;a_{8}=\varepsilon ,\;\;a_{9}=-\mu ,\\ a_{10}=-\left(\alpha +\mu \right),\;\;b_{1}=-\gamma ,\;\;b_{2}=\gamma . \end{array}$$

The characteristic equation of system (4) is

(6) λ4+A3λ3+A2λ2+A1λ+A0  +(B3λ3+B2λ2+B1λ+B0)e−λτ=0,

where

(7) A0=a1a6a9a10+a8a10(a3a5−a1a7),A1=a7a8a10+a8(a1a7−a3a5)−a1a6(a9+a10)−a9a10(a1+a6),A2=a1a6+a9a10−a7a8+(a1+a6)(a9+a10),A3=−(a1+a6+a9+a10),B0=a10b1(a1a6−a2a5),B1=(a2a5−a1a6−a1a10−a6a10)b1,B2=(a1+a6+a10)b1,B3=−b1.

For *τ* = 0, (6) reduces to

(8) $$\begin{array}{c} \lambda^{4}+A_{13}\lambda^{3}+A_{12}\lambda^{2}+A_{11}\lambda +A_{10}=0, \end{array}$$

where

(9) $$\begin{array}{c} A_{10}=A_{0}+B_{0},\;\;A_{11}=A_{1}+B_{1},\\ A_{12}=A_{2}+B_{2},\;\;A_{13}=A_{3}+B_{3}. \end{array}$$

Obviously, if condition (*H*_1_) (10) holds, *D*_∗_ is locally asymptotically stable in the absence of delay according to the Routh-Hurwitz criterion. One has

(10) Det1=A13>0,Det2=|A131A11A12|>0,Det3=|A1310A11A12A130A10A11|>0,Det4=|A13100A11A12A1310A10A11A12000A10|>0.

For *τ* > 0, let *λ* = *iω*  (*ω* > 0) be the root of (6). Then, we get

(11) (B1ω−B3ω3)sinτω+(B0−B2ω2)cos⁡τω =A2ω2−ω4−A0,(B1ω−B3ω3)cos⁡τω−(B0−B2ω2)sinτω =A3ω3−A1ω,

from which one can get

(12) $$\begin{array}{c} \omega^{8}+c_{3}\omega^{6}+c_{2}\omega^{4}+c_{1}\omega^{2}+c_{0}=0, \end{array}$$

with

(13) c0=A02−B02,c1=A12−B12−2A0A2+2B0B2,c2=A22−B22+2A0−2A1A3+2B1B3,c3=A32−B32−2A2.

Let *ω* ^2^ = *v*; then, (12) becomes

(14) $$\begin{array}{c} v^{4}+c_{3}v^{3}+c_{2}v^{2}+c_{1}v+c_{0}=0. \end{array}$$

In order to give the main results in this paper, we make the following assumption.

(*H*_2_) Equation (14) has at least one positive root.

If condition (*H*_2_) holds, we know that (12) has at least a positive root *ω*_0_ such that (6) has a pair of purely imaginary roots ±*iω*_0_. For *ω*_0_, the corresponding critical value of the delay is

(15) $$\begin{array}{c} \tau_{0}=\frac{1}{\omega_{0}}\text{arccos}\frac{p_{6}\omega_{0}^{6}+p_{4}\omega_{0}^{4}+p_{2}\omega_{0}^{2}+p_{0}}{q_{6}\omega_{0}^{6}+q_{4}\omega_{0}^{4}+q_{2}\omega_{0}^{2}+q_{0}}, \end{array}$$

where

(16) $$\begin{array}{c} p_{0}=-A_{0}B_{0},\;\;p_{2}=A_{0}B_{2}-A_{1}B_{1}+A_{2}B_{0},\\ p_{4}=A_{1}B_{3}-A_{2}B_{2}+A_{3}B_{1}-B_{0},\\ p_{6}=B_{2}-A_{3}B_{3},\;\;q_{0}=B_{0}^{2},\;\;q_{2}=B_{1}^{2}-2B_{0}B_{2},\\ q_{4}=B_{2}^{2}-2B_{1}B_{3},\;\;q_{6}=B_{3}^{2}. \end{array}$$

Taking the derivative with respect to *τ* on both sides of (6), we obtain

(17) [dλdτ]−1=−4λ3+3A3λ2+2A2λ+A1λ(λ4+A3λ3+A2λ2+A1λ+A0)+3B3λ2+2B2λ+B1λ(B3λ3+B2λ2+B1λ+B0).

Thus,

(18) Re[dλdτ]τ=τ0−1 =f′(v∗)B32ω06+(B22−2B1B3)ω04+(B12−2B0B2)ω02+B02,

where *v*_∗_ = *ω*_0_^2^ and *f*(*v*) = *v* ^4^ + *c*_3_ *v* ^3^ + *c*_2_ *v* ^2^ + *c*_1_ *v* + *c*_0_.

Thus, if condition (*H*_3_) *f*′(*v*_∗_) ≠ 0, then *Re*[*dλ*/*dτ*]_*τ*=*τ*_0__^−1^ ≠ 0. According to the Hopf bifurcation theorem in [16], we have the following results.

**Theorem 1 .**

If conditions (*H*_1_)–(*H*_3_) hold, then the positive equilibrium *D*_∗_(*S*_∗_, *L*_∗_, *B*_∗_, *R*_∗_) of system (2) is asymptotically stable for *τ* ∈ [0, *τ*_0_) and system (2) undergoes a Hopf bifurcation at the positive equilibrium *D*_∗_(*S*_∗_, *L*_∗_, *B*_∗_, *R*_∗_) when *τ* = *τ*_0_.

## 3. Properties of the Hopf Bifurcation

Let *u*_1_(*t*) = *S*(*t*) − *S*_∗_, *u*_2_(*t*) = *L*(*t*) − *L*_∗_, *u*_3_(*t*) = *B*(*t*) − *B*_∗_, *u*_4_(*t*) = *R*(*t*) − *R*_∗_, and *τ* = *τ*_0_ + *μ*, *μ* ∈ *R*, and normalize the delay by *t* → (*t*/*τ*). Then, system (2) can be transformed into a functional differential equation (PDF) as

(19) $$\begin{array}{c} \dot{u}\left(t\right)=L_{\mu }u_{t}+F\left(\mu ,u_{t}\right), \end{array}$$

where

(20) ut=(u1(t),u2(t),u3(t),u4(t))T∈C=  C([−1,0],R4),Lμϕ=(τ0+μ)(A′ϕ(0)+B′ϕ(−1)),F(μ,ut)(τ0+μ)(−β(ϕ1(0)ϕ2(0)+ϕ1(0)ϕ3(0))β(ϕ1(0)ϕ2(0)+ϕ1(0)ϕ3(0))00),

where

(21) $$\begin{array}{c} A^{\prime }=\left(\begin{array}{cccc} a_{1} & a_{2} & a_{3} & a_{4}\\ a_{5} & a_{6} & a_{7} & 0\\ 0 & a_{8} & a_{9} & 0\\ 0 & 0 & 0 & a_{10} \end{array}\right),\;\;B^{\prime }=\left(\begin{array}{cccc} 0 & 0 & 0 & 0\\ 0 & 0 & 0 & 0\\ 0 & 0 & b_{1} & 0\\ 0 & 0 & b_{2} & 0 \end{array}\right). \end{array}$$

By the Riesz representation theorem, there is a 4 × 4 matrix function with bounded variation components *η*(*θ*, *μ*), *θ* ∈ [−1,0] such that

(22) $$\begin{array}{c} L_{\mu }\phi =\overset{0}{\underset{-1}{\int }}d\eta \left(\theta ,\mu \right)\phi \left(\theta \right),\;\phi \in C. \end{array}$$

In fact, we choose

(23) $$\begin{array}{c} \eta \left(\theta ,\mu \right)=\left(\tau_{0}+\mu \right)\left(A^{\prime }\delta \left(\theta \right)+B^{\prime }\delta \left(\theta +1\right)\right). \end{array}$$

For *ϕ* ∈ *C*([−1,0]), *R* ^4^), we define

(24) A(μ)ϕ={dϕ(θ)dθ,−1≤θ<0,∫−10dη(θ,μ)ϕ(θ),θ=0,R(μ)ϕ={0,−1≤θ<0,F(μ,ϕ),θ=0.

Then, system (19) is equivalent to the following form:

(25) $$\begin{array}{c} \dot{u}\left(t\right)=A\left(\mu \right)u_{t}+R\left(\mu \right)u_{t}. \end{array}$$

Next, we define the adjoint operator A* of *A* as

(26) $$\begin{array}{c} A^{*}\left(\mu \right)s=\{\begin{array}{cc} -\frac{d\varphi \left(s\right)}{ds}, & 0<s\leq 1,\\ \overset{0}{\underset{-1}{\int }}d\eta^{T}\left(s,\mu \right)\varphi \left(-s\right), & s=0 \end{array} \end{array}$$

and a bilinear inner product as

(27) $$\begin{array}{c} \langle \varphi ,\phi \rangle =\overset{-}{\varphi }\left(0\right)\phi \left(0\right)-\overset{0}{\underset{\theta =-1}{\int }}\overset{\theta }{\underset{\xi =0}{\int }}\overset{-}{\varphi }\left(\xi -\theta \right)d\eta \left(\theta \right)\phi \left(\xi \right)d\xi , \end{array}$$

where *η*(*θ*) = *η*(*θ*, 0).

Let *ρ*(*θ*) = (1, *ρ*_2_, *ρ*_3_, *ρ*_4_)^*T*^ *e* ^*iτ*_0_*ω*_0_*θ*^ be the eigenvector of *A*(0) corresponding to the eigenvalue +*iτ*_0_ *ω*_0_ and let ρ*(*θ*) = *D*(1, *ρ*_2_*, *ρ*_3_*, *ρ*_4_*)*e* ^*iτ*_0_*ω*_0_*s*^ be the eigenvector of A*(0) corresponding to the eigenvalue −*iτ*_0_ *ω*_0_. Then, from the definition of *A*(0) and A*(0), we obtain

(28) $$\begin{array}{c} \rho_{2}=\frac{a_{5}+a_{7}\rho_{3}}{\tau_{0}\omega_{0}-a_{6}},\;\;\rho_{3}=\frac{a_{5}a_{8}}{\left(i\omega_{0}-a_{6}\right)\left(i\omega_{0}-a_{9}-b_{1}e^{-i\tau_{0}\omega_{0}}\right)},\\ \rho_{4}=\frac{i\omega_{0}-a_{1}-a_{2}\rho_{2}-a_{3}\rho_{3}}{a_{4}},\\ \rho_{2}^{*}=-\frac{i\omega_{0}+a_{1}}{a_{5}},\;\;\rho_{3}^{*}=-\frac{a_{2}+\left(i\omega_{0}+a_{6}\right)\rho_{2}^{*}}{a_{8}},\\ \rho_{4}^{*}=-\frac{a_{3}+a_{7}\rho_{2}^{*}+\left(i\omega_{0}+a_{9}+b_{1}e^{i\tau_{0}\omega_{0}}\right)\rho_{2}^{*}}{b_{2}e^{i\tau_{0}\omega_{0}}}. \end{array}$$

From (27), we can get

(29) 〈q∗(s),q(θ)〉=ρ−[1+ρ2ρ−2∗+ρ3ρ−3∗+ρ4ρ−4∗+τ0ρ3e−iτ0ω0(b1ρ−3∗+b2ρ−4∗)].

Then, we choose

(30) D−=[1+ρ2ρ−2∗+ρ3ρ−3∗+ρ4ρ−4∗+τ0ρ3e−iτ0ω0(b1ρ−3∗+b2ρ−4∗)]−1

such that 〈ρ*, *ρ*〉 = 1 and $\langle \rho^{*},\overset{-}{\rho }\rangle =0$.

Following the algorithms given in [16] and using similar computation process in [17], we can get the following important coefficients:

(31) g20  =2τ0D−β(ρ−2∗ρ3−ρ2),g11=τ0D−β(ρ−2∗(ρ3+ρ−3)−(ρ2+ρ−2)),g02=  2τ0D−β(ρ−2∗ρ−3−ρ−2),g21=2τ0D−β(ρ−2∗(W11(1)(0)ρ3+12W20(1)(0)ρ−3+W11(3)(0)+12W20(3))−(W11(1)(0)ρ2+12W20(1)(0)ρ−2+W11(2)(0)+12W20(2))),

with

(32) W20(θ)=ig20ρ(0)τ0ω0eiτ0ω0θ+ig−02ρ−(0)3τ0ω0e−iτ0ω0θ+E1e2iτ0ω0θ,W11(θ)=−ig11ρ(0)τ0ω0eiτ0ω0θ+ig−11ρ−(0)τ0ω0e−iτ0ω0θ+E2,

where *E*_1_ and *E*_2_ can be determined by the following equations, respectively,

(33) (2iω0−a1−a2−a3−a4−a52iω0−a6−a700−a82iω0−a9−b1e−2iτ0ω0000−b2e−2iτ0ω02iω0−a10) =2(E1(1)E1(2)00),(a1a2a3a4a5a6a700a8a9+b1000b2a10)E2=−(E2(1)E2(2)00),

with

(34) $$\begin{array}{c} E_{1}^{\left(1\right)}=-\beta \rho_{2},\;\;E_{1}^{\left(2\right)}=\beta \rho_{2},\\ E_{2}^{\left(1\right)}=-\beta \left(\rho_{2}+{\overset{-}{\rho }}_{2}\right),\;\;E_{2}^{\left(2\right)}=\beta \left(\rho_{3}+{\overset{-}{\rho }}_{3}\right). \end{array}$$

Then, we can get the following coefficients:

(35) C1(0)=i2τ0ω0(g11g20−2|g11|2−|g02|23)+g212,μ2=−Re{C1(0)}Re{λ′(τ0)},β2=2Re{C1(0)},T2=−Im⁡{C1(0)}+μ2Im⁡{λ′(τ0)}τ0ω0.

In conclusion, we have the following results.

**Theorem 2 .**

For system (2), If *μ*_2_ > 0  (*μ*_2_ < 0), then the Hopf bifurcation is supercritical (subcritical). If *β*_2_ < 0  (*β*_2_ > 0), then the bifurcating periodic solutions are stable (unstable). If *T*_2_ > 0  (*T*_2_ < 0), then the period of the bifurcating periodic solutions increases (decreases).

## 4. Numerical Simulation

**Figure 1:**
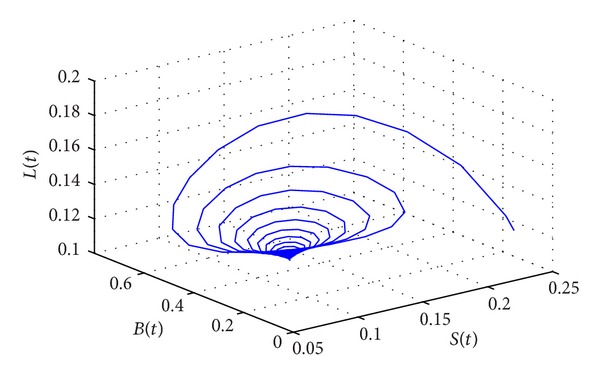
The phase plot of states *S*, *L*, and *B* for *τ* = 55.2386 < *τ*_0_ = 58.5472.

**Figure 2:**
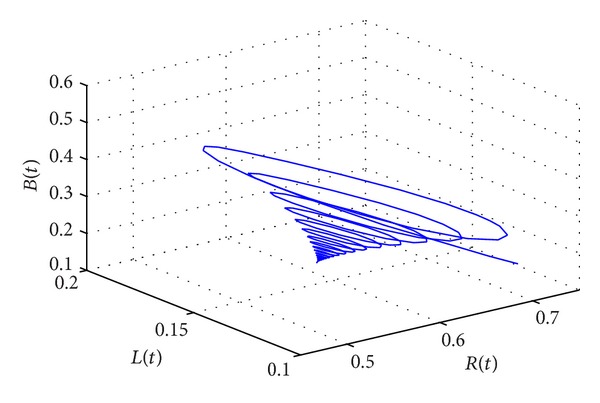
The phase plot of states *L*, *B*, and *R* for *τ* = 55.2386 < *τ*_0_ = 58.5472.

**Figure 3:**
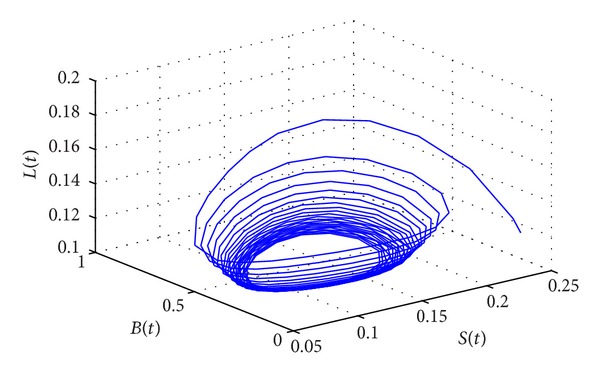
The phase plot of states *S*, *L*, and *B* for *τ* = 60.5386 > *τ*_0_ = 58.5472.

**Figure 4:**
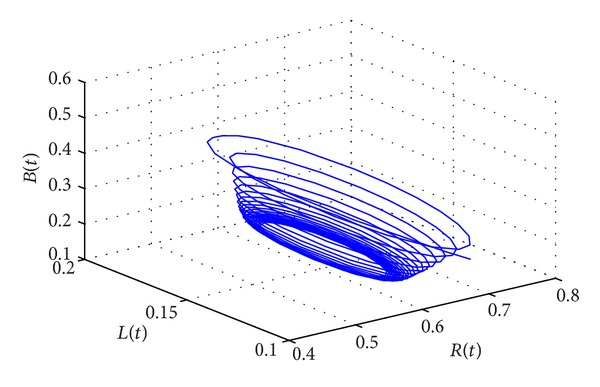
The phase plot of states *L*, *B*, and *R* for *τ* = 60.5386 > *τ*_0_ = 58.5472.

In this section, we present a numerical example to justify the theoretical results above. We use the same coefficients as those used in [7] and consider the following system:

(36) dS(t)dt=  0.001−0.15S(t)(L(t)+B(t))−0.001S(t)+0.01R(t),dL(t)dt=  0.15S(t)(L(t)+B(t))−0.05L(t)−0.001L(t),dB(t)dt=  0.05L(t)−0.02B(t−τ)−0.001B(t),dR(t)dt=  0.02B(t−τ)−0.01R(t)−0.001R(t),

from which we obtain the basic reproduction number *R*_0_ = 9.944 > 1 and the unique positive equilibrium *D*_∗_(0.1006,0.1160,0.2762,0.5022) of system (36). Further, we obtain *ω*_0_ = 2.3091, *τ*_0_ = 58.5472. According to Theorem 1 in Section 2, we know that, when *τ* ∈ [0,58.5472), the positive equilibrium *D*_∗_(0.1006,0.1160,0.2762,0.5022) of system (36) is locally asymptotically stable. As can be seen from Figures 1 and 2, when *τ* = 55.2386 < *τ*_0_, the positive equilibrium *D*_∗_(0.1006,0.1160,0.2762,0.5022) of system (36) is locally asymptotically stable. However, when the delay passes through the critical value of *τ*_0_ = 58.5472, the positive equilibrium *D*_∗_ will lose stability and a Hopf bifurcation occurs. This property can be illustrated by Figures 3 and 4.

In addition, by some complex computations and from (35), we obtain *μ*_2_ = −0.8143 < 0, *β*_2_ = −0.9001 < 0, and *T*_2_ = 0.0466 > 0. Therefore, we can conclude that the Hopf bifurcation of system (36) is subcritical, the bifurcating periodic solutions are stable, and the period of the periodic solutions decreases according to Theorem 2 in Section 3.

## 5. Conclusions

A delayed SLBRS computer virus model has been studied in this paper based on the model proposed in [7]. Compared with literature [7], we mainly consider the effect of the time delay due to the period that the computers use antivirus software to clean the virus on the model. The main results are presented in terms of local stability and local Hopf bifurcation. Sufficient conditions for local stability and existence of local Hopf bifurcation are obtained by analyzing the distribution of the roots of the associated characteristic equation. It has been shown that when the delay is suitably small (*τ* < *τ*_0_), the computer virus model is locally asymptotically stable. In such case, the propagation of the computer virus can be easily controlled. However, once the delay passes though the critical value *τ*_0_, a Hopf bifurcation occurs and a family of periodic solutions bifurcates from the positive equilibrium of the model. This is not welcome in networks because the computer virus will be out of control in this case. Further, direction and stability of the Hopf bifurcation have also been determined by using the normal form method and center manifold theory. Numerical simulations are also included to verify the analytical predictions.
